# Risks in Surrogacy Considering the Embryo: From the Preimplantation to the Gestational and Neonatal Period

**DOI:** 10.1155/2018/6287507

**Published:** 2018-07-17

**Authors:** M. Simopoulou, K. Sfakianoudis, P. Tsioulou, A. Rapani, G. Anifandis, A. Pantou, S. Bolaris, P. Bakas, E. Deligeoroglou, K. Pantos, M. Koutsilieris

**Affiliations:** ^1^Department of Physiology, Medical School, National and Kapodistrian University of Athens, Greece; ^2^Assisted Conception Unit, 2^nd^ Department of Obstetrics and Gynecology, Aretaieion Hospital, Medical School, National and Kapodistrian University of Athens, Greece; ^3^Centre for Human Reproduction, Genesis Athens Clinic, Greece; ^4^Department of Histology and Embryology, Faculty of Medicine, University of Thessaly, Greece; ^5^Assisted Conception Unit, General-Maternity District Hospital “Elena Venizelou”, Greece

## Abstract

Surrogacy is an assisted reproduction-based approach in which the intended parents assign the gestation and birth to another woman called the surrogate mother. The drivers of surrogacy refer largely to infertility, medical conditions, same-sex couples' parenting, and cases of diversity regarding sexual identity and orientation. Surrogacy consists of a valid option for a variety of conditions or circumstances ranging from medical to social reasons. However, surrogacy may be associated with risks during the preimplantation, prenatal, and neonatal period. It became obvious during the exhaustive literature research that data on surrogacy and its association with factors specific to the IVF practice and the options available were not fully represented. Could it be that surrogacy management adds another level of complexity to the process from the ovarian stimulation, the subsequent IVF cycle, and the techniques employed within the IVF and the Genetic Laboratory to the fetal, perinatal, and neonatal period? This work emphasizes the risks associated with surrogacy with respect to the preimplantation embryo, the fetus, and the infant. Moreover, it further calls for larger studies reporting on surrogacy and comparing the surrogate management to that of the routine IVF patient in order to avoid suboptimal management of a surrogate cycle. This is of particular importance in light of the fact that the surrogate cycle may include not only the surrogate but also the egg donor, sperm donor, and the commissioning couple or single person.

## 1. Introduction

Over the last decades, infertility has become a matter that the majority of infertile couples choose to address. Surrogacy has revolutionized the standing options within the assisted reproduction field, enabling the gestation and birth of child by another woman—the surrogate mother—relinquishing the child after birth to the commissioning parents [1–3].

The first type of surrogacy is the traditional (or genetic) surrogacy, in which the father's sperm or the donor's sperm is naturally or artificially employed to inseminate the surrogate's oocyte (homologous IVF). This approach leads to an embryo genetically linked to the surrogate. The second type is the gestational (or host) surrogacy. In this case the implanted embryo shares no genetic link with the surrogate mother. In gestational surrogacy, the embryo results from heterologous IVF employing the intended parents' gametes, or the intended father's sperm and donor's oocyte (not the surrogate's), or the donor's sperm and the intended mother's oocyte. Alternatively, the embryo could be donated [1–4].

Infertility, medical conditions, diversities regarding sexual identity and orientation, and matters of social nature reflect the basic drivers behind patients' decision to pursue surrogacy. Women with a severe Müllerian anomaly or a congenital absence of uterus and/or vagina are usual candidates for surrogacy. The condition of Mayer-Rokitansky-Küster-Hauser Syndrome characterized by a female genotype and phenotype and accompanied with a congenital aplasia of the uterus and the vagina [5–7] represents another category of patients requiring surrogacy. Further to the above, Complete Androgen Insensitivity Syndrome (CAIS syndrome) where the uterus and ovaries are absent [8, 9], as well as women who have undergone hysterectomy, presents as only few of the cases where surrogacy is imperative and women may choose this as a path to parenthood. Gestational surrogacy is also recommended in cases of Turner's Syndrome due to the known cardiac and medical complications in these patients [10]. Furthermore, surrogacy may present as an option for women with recurrent miscarriages or unidentified failure of infertility treatment [6]. Heart or renal disease and severe Rhesus isoimmunization during pregnancy are valid reasons for the mother to avoid such high risk conditions and hence constitute solid grounds in opting for surrogacy [9]. Other indications for surrogacy are maternal medication for specific disease treatment that could potentially promote embryo's teratogenesis [11] or even severe genetic problems related to the intended parents [12]. Social reasons towards optional surrogacy may correspond to highly driven career women and the stress experienced by the intended mother regarding the physical changes and the discomfort associated with her perception on pregnancy [13]. Finally, surrogacy may fulfill same-sex couples' or even a single parent's desire for a genetically linked family [14, 15]. It is evident that the option of surrogacy corresponds to a wide range of drivers, extending beyond strictly medical reasons especially in today's era.

The majority of surrogates report that the main incentive is altruism, as surrogacy reinforces their self-esteem, despite the fact that financial gain may also be a major consideration. In many countries, any payment to gestational carriers is legally prohibited, solely allowing some financial aid only for pregnancy-related expenses [11, 16]. Specifically, altruistic surrogacy is adopted in England, in many states of United States, and in Australia, while commercial surrogacy is permitted in India, Ukraine, and California. On the other hand, surrogacy is not allowed in Germany, Sweden, Norway, and Italy [13].

Prior to initiating procedures, certain factors should be investigated and valued to ascertain safe outcome for both the surrogate and the embryo. The surrogate should undergo medical examinations and thorough psychological assessment, in order to be considered suitable. The psychological assessment evaluates the surrogate's ability to emotionally sustain gestation and delivery. It has been reported that her status throughout the gestation could affect the child's individuality and psychological wellbeing [3]. The age of the surrogate should range from 21 to <35 years for traditional surrogacy and to <45 years for gestational surrogacy [12], and her reproductive history profile should include at least one previous uncomplicated pregnancy, while not exceeding 5 deliveries or 3 caesarean sections. However, there have been reports on surrogates of advanced age for whom a court decision may allow their involvement, for instance, in cases when the surrogate is a family member, possibly the mother of the commissioning woman. The older surrogate has been reported by the media to be a 67-year-old woman serving as a surrogate for her daughter who developed uterine cancer leading to hysterectomy. Any lifestyle that could compromise the infant's health is barred and a legally bound contract between the commissioning parents and the surrogate must be signed, ascertaining all the aforementioned conditions [3].

To date surrogacy may not present a straightforward alternative among women of reproductive age. In an interesting study, when presented with the option, women responded they would rather opt for uterus transplantation instead, with a percentage of 80% vs 47% [17]. However, we should take into account that in Sweden—where the data came from—surrogacy is not an available option according to Swedish legislation. Laws and practices regarding surrogacy vary, highlighting the controversial nature of the issue, giving rise to numerous legal and ethical considerations [14]. This may be significantly attributed to surrogacy's association with various risks during the preimplantation, prenatal, and neonatal period. The results of surrogacy seem to be satisfying and promising, with a reported rate of up to 60% live births, as surrogate women tend to be fertile and young [6].

This article highlights the challenges and considerations associated with surrogacy. It uniquely brings to literature the respective associations regarding the preimplantation embryo, the fetus, and the infant. When dealing with a surrogate cycle within the IVF set-up, is it possible that the urgency for the cycle, in order to secure an optimal result, compromises its management? Could these pregnancies, being widely characterized as “precious,” result in choices and practices that ensure the highest percentages of success? Extrapolating on that concept, could these choices be selected on nonmedical grounds and hence pose a risk? Is it possible that we lean towards a hyperbole when surrogacy is the case?

## 2. Risk Factors Regarding the Preimplantation Embryo of a Surrogate Cycle

Surrogacy goes hand in hand with IVF treatment and every aspect that this entails. A surrogacy cycle within the IVF set-up includes superovulation, oocyte retrieval, fertilization techniques, embryo culture, embryo selection, embryo transfer, and possibly cryopreservation. It is evident that surrogacy cycles require the services of IVF irrespectively of infertility etiology. The fact that these embryos are created within the IVF set-up may leave room for further manipulation of the embryo. Extended culture to the blastocyst level may represent a straightforward example as it may be believed to secure better implantation potential [18]. Further to that point, these embryos may be subjected to genetic testing in the form of Preimplantation Genetic Screening (PGS). PGS may be employed in order to enhance and enrich selection criteria and identify the embryos carrying a balanced chromosomal complement, thus securing a healthy pregnancy [7, 19]. In this section we highlight the negative implications related to the fact that surrogate babies are in fact IVF babies.

### 2.1. Risk Factors Related to Controlled Ovarian Stimulation

Both embryo manipulation and environmental factors within the IVF laboratory set-up may allow for epigenetic changes during the first stages of embryo development. Under epigenetic influence, the control of gene expression through DNA methylation, histone modification, and miRNA could be altered [20]. These modifications are heritable despite the fact that they do not alter DNA sequences [21]. With respect to a surrogate IVF cycle, the superovulation regime is applied either on the commissioning mother in cases of autologous surrogacy (own oocytes) or on the oocyte donor or on the actual surrogate. At any rate, it is understandable for the desired oocyte yield to be high.

Epigenetic changes could occur due to Exogenous Gonadotropins (EGs) exposition. EGs are administered to the ovary to a secure successful superovulation regime through controlled ovarian stimulation (COS). It has been proposed that EGs may contribute to epigenetic changes in four imprinted genes,* peg1, kcnq1ot1, zac, *and* h19 *[18], and may impair oocyte and embryo development [22]. The strictly clinical nature of IVF does not allow for any attempt to thoroughly examine the preimplantation embryo on an epigenetic assessment level, as these embryos are destined for embryo transfer and/or cryopreservation. However, the study by Ventura-Lunca et al. demonstrated that these imprinted genes are associated with fetal growth retardation and issues regarding placental development [18]. Therefore, one could extrapolate that these detrimental epigenetic changes exert a detrimental effect on the preimplantation stage of development. Whether the defects on* peg1* gene and the methylation of* h19—*during the preimplantation period—are associated with superovulation, the patient's age, and the delayed oocyte maturation or if they were inherited, studies in human models reveal unclear conclusions. The imprinting defects involved may lead to clinical implications in ART, such as failure of the embryo to implant, spontaneous abortion, and/or fetal growth retardation attributed to dysfunctional placentas [23]. The data available should be further and thoroughly examined prior to conquering on the true effects of COS.

### 2.2. Risk Factors Related to ICSI Practice

In a surrogate cycle, aspiring to secure the highest fertilization rates, ICSI (Intracytoplasmic sperm injection) may be selected as the method of choice. On the grounds that fertilization results on ICSI are reported to be higher than standard IVF [24, 25], often this seems to be a welcomed approach for both patients and practitioners. However, is it safe to extrapolate that surrogacy cycles within the IVF practice are associated with higher percentages of ICSI practice? The insemination technique of ICSI may be related to further impairment of the embryo due to its invasive nature. In comparison to the standard IVF insemination technique, ICSI is a practice that overturns natural selection. An example of that is the override of the physiological sperm processes involved during fertilization, for instance, acrosomic reaction [26]. Selecting the most adequate sperm for ICSI based on morphology comes with great responsibility, since in vivo the procedure of insemination is performed via strict natural selection criteria. In this way, spermatozoa with decimated mobility or increased abnormal morphology may be employed during ICSI in cases of male factor infertility and thus lead to higher risk of de novo chromosomal anomalies in the ICSI offspring. Studies in mouse models observed that male ICSI offsprings with DNA fragmented sperm had reduced fertility potential [22]. Undoubtedly, ICSI is considered a safe, efficient, and routinely employed technique of insemination that has not been particularly associated with increased chromosomal or congenital abnormalities [24]. However, on account of the fact that ICSI practice has emerged in the late 90s [26] the correlation of ICSI practice and the offsprings' wellbeing has to be further evaluated to delineate whether the procedure or the couple's genetic background could be accountable for any future trends or observations.

### 2.3. Risk Factors Related to Embryo Culture

Aiming to secure the highest implantation potential of embryos produced in an IVF laboratory and included in a surrogate cycle, it is common to opt for blastocyst culture. However, bypassing all the benefits associated with this practice [27], the hazardous or ambiguous results associated with prolonged culture and its effect on the preimplantation embryo physiology and epigenetics have been extensively argued [28]. To date and to our knowledge, a study related to blastocyst culture and surrogacy has yet to be published. Nonetheless, our extensive clinical experience and data mined from available published studies support that blastocyst culture appears to be the culture method of choice when managing a surrogate cycle. Various conclusions could be extrapolated regarding the effect of media and culture conditions on embryonic development and epigenetics [29]. Several studies advocate that culture medium may be responsible for a variety of detrimental trends, namely: abnormal implantation, low implantation rate, disorders in developmental pace, low embryo quality, and reduced trophoblast development, as well as embryo cell number and hatching ability [18, 30, 31]. An allegedly, simple, and justified change in media formulation, such as inclusion of serum, could lead to neonatal implications as shown in animals [31], while oxygen concentration has been reported to affect embryo metabolism, protein synthesis, and function [18]. Efforts are still focused on formulating and proving the optimal media consistency for human embryo culture, as mimicking and even improving the in vivo conditions is an ongoing process.

### 2.4. Risk Factors Related to Embryo Manipulation

In the set-up of ART, transferring a euploid embryo to the surrogate mother is of paramount importance. This could ensure that the possibility of miscarriage, termination of pregnancy, or live birth related to a compatible with gestation disorder is minimized. In case of chromosomal abnormalities and/or monogenic disorders, Preimplantation Genetic Diagnosis and Screening (PGD/PGS) excavate monogenic diseases and chromosomal abnormalities, numerical or structural, leading to the best embryo selection [7, 19, 32]. It is not uncommon for surrogacy to be proposed as the optimal approach instead of PDG/PGS application, in cases of patients with recurrent miscarriages or with a reproductive history of autoimmune loss of pregnancy [33]. Given the option, it is possible that the commissioning couples decide to further subject the embryos—destined to be transferred to a surrogate uterus—to PGS on the grounds of acquiring more information on their genetic profile [7]. The further embryo manipulation may be opted for in order to enhance the selection of embryos to be transferred and therefore increase the pregnancy success rate [7]. In addition to that, one must not fail to report on the possibility that PGS may be requested and performed not solely on the grounds of selection criteria to enhance success rates, but aiming to select the embryo of “choice” entering a grey and dangerous territory of eugenics. Is it possible that within the set-up of IVF and surrogacy such practices are promoted? If so, we should thoroughly weigh the advantages and disadvantages of such practice, report on the benefits ensured by the additional invasive manipulation the embryos are subjected to, and most importantly ponder on the bioethical questions raised [16].

Various embryo biopsy strategies have been suggested, such as blastomere biopsy at the cleavage stage, trophectoderm biopsy at the blastocyst stage, polar body, and finally morula biopsy. Ensuring a careful embryo manipulation during biopsy to maintain its viability is pivotal [32]. However, one should never fail to recall that this still remains a highly invasive process associated with negative effects in animal studies. Biopsy of 1-2 blastomeres at the cleavage stage does not exert a negative effect on the further development of the embryo [33]. On the other hand, increased body weight coupled with impaired acoustic habituation in male mice offsprings has been suggested to be related to protein alteration as a result of PGS biopsy [22]. These represent just a few findings related to PGS application in animal model studies. The argument remains whether any adverse obstetric and neonatal outcomes could be attributed to biopsies performed for PGD or as hypothesized be strictly a result of de novo alterations or the parental profile contribution. This should be carefully acknowledged in cases of surrogacy where PGS is applied. Subsequently, in these cases vitrification is inevitable as it goes hand in hand with blastocyst biopsy. This approach is required in order to secure the appropriate time required for the genetic analysis to be performed. Therefore, embryo transfer is ensued at a later stage. Vitrification has revolutionized the application of PGD/PGS, allowing for complex and time consuming genetic analysis to be performed, offering results on the whole chromosomal complement of the embryo tested [34]. Cryopreservation of embryos or blastocysts is considered to have no major genetic or epigenetic risks [22, 35]. Partial correction of epigenetic changes that may occur during vitrification in oocytes or in early cleavage embryos is attributed to specific mechanisms, while in developing blastocysts it completely disappears [21]. Embryo manipulation should be carefully considered and ideally employed on valid grounds referring to medical etiology and not patient's desire. Embryo biopsy for PGS and subsequent vitrification should both be carefully considered in cases of surrogacy, where IVF services are strictly employed to enable surrogacy procedures. Having access to embryos created in the IVF laboratory should not always translate to using it. Invasive practices involve mechanisms that have not been entirely delineated yet, and hence they remain unpredictable.

### 2.5. Risk Factors Related to the Embryo Transfer Procedure

The number of the embryos transferred is characterized by controversy and debate. Numerous studies support the elective single embryo transfer (eSET), especially in cases of surrogacy, as the most efficient approach to limit multiple gestation and preterm birth, which are both accompanied by adverse perinatal and neonatal outcomes [4, 7, 36]. The elective single embryo transfer is embraced universally as the optimal method associated with the best perinatal and neonatal outcomes. However, the question raised is the following: could the cohort of preimplantation embryos produced for a surrogacy cycle be subjected to prolonged culture, in order to enhance selection of the best single embryo and enable eSET? The risks associated with prolonged culture should be addressed prior to applying this practice [28]. Further to that, PGS has been proposed to enhance and secure the eSET approach. However, another level of complexity to manipulating these embryos should be accounted for. This hypothesis may be contradicted; it is however imperative for this to be thoroughly examined. On the other hand, eSET may not be solely linked to prolonged culture or PGS application. Recently, the combination of time-lapse imaging with morphological parameters has claimed to revolutionize embryo selection as it may contribute by identifying aneuploid embryos avoiding detrimental effects on the embryo. On the same concept, the goal remains to allow for true continuous culture and evade embryo culture disruption. In this context, time-lapse technology could assist by minimizing events of epigenetic changes regarding the preimplantation [37]. Minimizing the already invasive nature of IVF is considered to be the holy grail of embryology with time-lapse imaging presenting as the first promising attempt [38].

## 3. Risk Factors Related to the Gestation and the Fetus

In contradiction to natural conceptions, pregnancies deriving from ART cycles—including surrogacy cycles—may be related to increased risk of perinatal complications. It has been indicated that the perinatal outcomes of gestational surrogacy in comparison to autologous IVF report no major increase in the risks of preterm birth, live birth rate, and congenital anomalies [4]. In addition to that, it was contemplated that oocyte donation demonstrates poor fetal immunological adjustment to allogeneic antigen. As a result, gestational surrogacy appears to be associated with a higher risk of hypertensive disorders than autologous IVF [16]. Prior exposure of the embryo to culture medium in the IVF set-up could contribute to perinatal complications as well, such as unbalanced fetal placenta development, abnormal fetal growth, and metabolic responses [18]. An increased systolic blood pressure in 21-week-old mice with previous IVF culture has been indicated, as well as a minor anxiety, psychomotor activity, and special memory in rat embryos [31].

In order to increase implantation rates, the method of multiple embryos transfer is opted for in some IVF cases. This practice is also adopted and perhaps even heightened in the cases of surrogacy, resulting in multiple gestations with the obstetric and perinatal complications that these may entail [14]. IVF-surrogates may present with a lower incidence of third trimester's complications, such as pregnancy-induced hypertension, placenta praevia and abruption, diabetes mellitus, and hemorrhage, irrespective of whether it was a multiple gestation or singleton, in comparison to women subjected to standard IVF. However, it was evident that IVF surrogacy with multiple gestations is associated with increased risk of preeclampsia, postpartum hemorrhage, hysterectomy, and gestational diabetes [39]. In addition to the above, multiple pregnancies are related to higher risk of hyperemesis and anemia [14]. In light of the above, multiple gestations—especially in surrogate cycles—should be avoided; thus supporting eSET practice is highly recommended [7]. In cases of multiple gestations associated with challenging obstetric complications, selective feticide may be an option. Nevertheless, it constitutes a risky alternative. Performance of fetal intracardiac injection of potassium chloride for selective feticide has been re-evaluated due to the high risk it presents to the fetus not subjected to the procedure [40]. The use of radiofrequency ablation interrupting blood flow to the selected fetus is considered as potentially being the most effective option for any gestational age [41]. It is argued whether parents have the right to decide for selective feticide, as there is more interaction between the surrogate's body and the developing fetuses.

The special conditions and the uniqueness characterizing surrogacy, the relationship, and the expectations of the commissioning couple/person towards the surrogate could complicate management. This is a situation understandably lacking control, a condition which certainly may create the basis for a pregnancy associated with an extra level of stress factors [3]. Exposure to maternal stressors during pregnancy activates the hypothalamus-pituitary-adrenal cortex system or hypothalamic–pituitary–adrenal (HPA) axis and sympathetic system as well, which provoke hormones' production, such as CRH (corticotrophin-releasing hormone), ACTH (adrenocorticotropin-releasing hormone), cortisol, adrenalin, and noradrenaline in maternal blood [42]. It is evident that maternal stress may affect the unborn baby through the secretion of mother's stress hormones (such as ACTH, CRH, prolactin, and oxytocin). The increased levels of the aforementioned hormones are associated with reduction of uteroplacental blood flow, leading to fetal growth restriction (IUGR: Intrauterine Growth Restriction) [42]. Interestingly, it has been evaluated via ultrasound examination that fetuses of highly anxious women at the 36 gestational weeks present increased bodily activity [42]. What is more, the high levels of hormones may indicate and contribute to automatic abortion, structural malformations, and preeclampsia. This may be the result of depression or anxiety during the first trimester, coupled with high levels of placental CRH and smaller head circumference, affecting brain growth [42]. Maternal hormones, like adrenalin, are produced in maternal blood in cases of fear and/or discomfort and provoke the stress' symptoms of tachycardia and breathe acceleration. In addition, maternal stress during the third trimester contributes to preterm uterine activity, leading to preterm delivery, while maternal stress in the first trimester may lead to low birth weight [43].

During the fetal period, the surrogate mother has to abide by all agreed limitations, as she is exposed to the same pregnancy risks as any pregnant woman. This means that she is vulnerable to ectopic pregnancy or even miscarriage [2]. Conformation to limitations indicates avoiding drugs or alcohol consumption. In this way, risks regarding structural and functional abnormalities which could lead to adulthood physical or mental defects are minimized. What is more, the surrogate mother—equally to any pregnant woman—should adopt an appropriate diet, as nutrition deficiency could permanently alter the development or function of a specific organ [42].

Without a doubt the psychological and emotional states of the surrogate play a pivotal role in the wellbeing of the fetus. Stemming from the behavior and/or the stress level of the surrogate, her state may translate biologically to deleterious intrinsic factors that affect the wellbeing and development of the fetus. Could it be that the possible lack of acceptance of the surrogate towards recognizing the embryo and fetus as her own and the possible lack of positive outlook of the pregnancy may present a risk to the development of the fetus? It is suggested that the gestational mother may contribute to fetal development, through epigenetics, microchimerism (cells are transferred between the fetus and mother through placenta), and transport of both antibodies and nutrients [1]. Fetal consciousness develops from the uterine to breastfeeding period and numerous physiological, emotional, or environmental messages affect its development. Consequently, maternal acceptance or rejection could be a stimuli imprinted in human cells. Medical evidence proves that increased stress hormones in maternal blood, such as adrenalin, penetrate placenta and invade to fetal blood, causing fetal rapid heartbeat or breathing acceleration [43].

Dar's interesting study examining all issues from medical to psychosocial and legal exploring data from a large surrogacy program reported an overall mean of 37,9 weeks of gestational age at birth on surrogacy cases and specifically 38,9 weeks for singletons and 35,8 weeks for multiple births [11]. These results seem to be comparable with previous studies. The maternal complication rate reported by Dar et al. was 9.8% which is considerably low due to the fact that surrogate mothers have a history of previous healthy pregnancies without any complications. Fetal anomalies in the same study are presented with a prevalence of only 1.8% possibly attributed to the extensive obstetric history check that the surrogates are subjected to or due to the fact that surrogacy is often enabled by oocyte donation [11]. Surrogate candidates are meticulously examined, their background investigation is thorough, and their medical profile may be often ideal. On the other hand, women who conceive naturally do not necessarily fulfill all the above prerequisites. Therefore, positive results related to surrogacy with respect to complications may be anticipated, contradicting the notion that complications and complexity may be heightened in surrogacy cycles.

## 4. Risk Factors Related to the Neonatal and the Period Thereafter

Whether factors related to the surrogate pregnancy find their way towards affecting the neonatal and the period following is a subject under investigation. Many studies propose that ART offsprings—and that extends to surrogacy cases—are prone to cardiovascular diseases, presenting with higher systolic and diastolic blood pressure, obesity resulting from insulin resistance and the impaired glucose metabolism, and thyroid dysfunction with high levels of thyroid-stimulating hormone (TSH) [22]. On the other hand, there are reports indicating that IVF techniques may not extend to burdened perinatal and neonatal complications. The study by Chian et al. 2008 examined 200 infants deriving from three different centers in Canada, born from vitrified oocytes, and concluded that vitrification had no effect on fetus and baby [44].

As mentioned above, the practice of multiple embryos included in the ET may result in multiple gestations often encountered in surrogacy cycles. These may result in preterm labour and delivery, in comparison to singleton [2, 5, 39]. Consequently, babies present with low birth weight or they fail to sustain perhaps even due to prematurity alone or accompanied by a deformity or abnormality [1]. Furthermore, newborns of multiple gestations may present with speech delays and developmental handicaps [5], as well as cerebral palsy [1]. What is more, prematurity, directly related to multiple gestations, contributes to congenital malformations and increased rates of caesarean sections, in comparison to singleton [39]. In contrast to the above, a follow-up on babies born through multiple or singleton IVF surrogacy showed that motor delays cease at the second year of their life [5]. On the other hand, singleton IVF-children present with no further physical anomalies, taking into consideration that defect embryos often fail to implant. This is in contrast to multiple gestations, which appear to be associated with low-birthweight infants and/or with minor heart and lung defects [45]. Multiple gestations associated with complications in surrogate pregnancies may be avoided by opting for eSET as discussed above and managed employing the practice of selective feticide. This, especially in complex cases, may entail a therapeutic nature by creating safer conditions for the surrogate's health as well for the infant to be. However, it is best to avoid reaching the point when it becomes a necessity and selective feticide becomes an option. The complications associated with its practice are numerous. Neurodevelopmental impairment, including cognitive, motor, and behavioral aspects, has been detected in 6.8% of the reported cases following selective feticide, while this finding appears to be more frequent in comparison to the general population [46].

The solution to challenges originating from multiple gestations is for the IVF set-up to promote further the practice of elective single embryo transfer to avoid multiple gestations and the considerable risks associated with them [7, 36]. The Ethics Committee of the American Society for Reproduction (ASRM) underlies the need for the gestational surrogate to be protected, by inclusively informing her regarding all the possible risks multiple pregnancies entail. On that concept, it becomes apparent that the final decision regarding the number of embryos to be transferred should be the surrogate's [14].

Nutrition of the surrogate is an important factor that could pose a risk. Insufficient nutrition during pregnancy plays an important role to the child's or even to the adult's health, as it may be responsible for the development of cardiovascular diseases, allergies, hypertension, diabetes, or either schizophrenia [42]. This is also confirmed by the Barker hypothesis, according to which the appearance of metabolic syndromes in adulthood may be attributed to the mother's malnutrition during pregnancy [18]. To extend this to the IVF environment, it has been shown that there is clear association between protein deficiency in embryo culture media and the child' birth weight [18, 19]. Information involving nutrition of the surrogate is scarce and difficult to control or record especially in reflection to perinatal data. Therefore, especially in light of the lack of knowledge, it is imperative to evaluate the mode and strength of the association between nutrition of the surrogate and respective implications on the children.

Stress levels of the surrogate during gestation could play a detrimental role. This exhaustive search did not identify studies reporting on whether maternal stress levels are higher during a surrogate pregnancy in comparison to nonsurrogate pregnancy. This fact may highlight a deficit in the literature. General population studies show a clear association between maternal stress and low birth weight or prematurity [42]. Neonatal studies on infants from highly anxious mothers recorded persistent crying during the first seven months of life and neonates characterized by irritability, irregular biological functions, and gripes. Later at the age of nine, these children were classified as overactive and poor sleepers [42, 43]. Prenatal maternal stress plays an important role to the infants' behavior, as studies observed that infants were categorized as antisocial and with low frustration threshold [43]. Ward's study evidenced a correlation between the development of childhood psychopathology and various prenatal conditions, such as maternal chronic or prenatal stress and anxiety, maternal acceptance of pregnancy, and some excessive physical reactions to pregnancy like vomiting [47]. Psychiatric observations showed that maternal stress during pregnancy plays a pivotal role to the appearance of Attention Deficit Hyperactivity Disorder (ADHD), schizophrenia, and depression. Characteristically, depressive adults have high levels of blood cortisol and CRH hormones [42].

Children born through gestational surrogacy are legally protected, through the anonymity of the donor and surrogate's data. However, the child has the right to be informed in a specific manner on the way that he/she was born and be informed of his/her origins in general [48]. Many studies record a positive child's reaction when information is released regarding the surrogacy, either traditional or gestational [49].

The science of prenatal and perinatal psychology reveals that every stimulus recorded to the child's consciousness significantly determines its behavior as an adult, both physical health and mental balance. Moreover, it defines the relationships that the child forms throughout life. In addition to that, many clinical studies assume that the embryo's conscience is formed during the intrauterine period and that the perception and the feelings of the surrogate during gestation may affect infant development majorly. Medical evidence supports the fact that various neurohormones are transferred from mother to fetus during pregnancy. These are pivotal for fetal brain development, normal neural system's function, and the future child's self-confidence and intelligence [43]. Good communication and feelings of acceptance act catalytically on the communication between mother and fetus and consequently contribute to important developmental aspects extending even to the child's speech ability [3].

It may be that trigger points regarding the psychological status of an expectant mother are the same between surrogates and nonsurrogates. If that can be safely hypothesized, then negative factors such as prenatal maternal stress, perceptions, and feelings will be expected to equally affect both groups at the same extent. However, is it equally safe to assume that such issues and detrimental effects will burden the infants of a surrogate pregnancy further? Additional studies are required to enrich our knowledge on such important issues on surrogacy. Could it be that a surrogate pregnancy, even though it is a product of consent and informed decision of the surrogate, may differ with respect to the feelings involved regarding a natural occurring desired pregnancy?

With respect to the psychological effect on the children born through surrogacy there are conflicting reports. Children born from a surrogate mother do not differ in their behavior [2, 3]. These offspring, at the age of two, seem to have no difficulties in their social integration and their cognitive and emotional development. Later, at the ages of three, seven, and ten, their psychological prosperity was found to be at the same levels as the other peer-to-peer children [3].

Another study examined the impact of surrogacy—genetically linked or not—on the children's psychological wellbeing during the first three years of their life, as well as during the preschool period at the age of seven. The results assessed family processes, such as warmth, communication among members, and conflict. The study concluded that, at ages of one, two, and three, children were overall unaware of the way they were born and family relationship appeared to be warmer and more enjoyable, in comparison to family processes regarding naturally conceived children. Later, at the age of seven, children presented a more positive relationship with their mothers, in contrast to natural conception families. The study reported that family structure, for instance, male same-sex or lesbian families, seems not to influence the children's psyche, as a positive quality of family relationship was evident [49]. In addition to this, a systematic review comparing children born through gestational surrogacy and those born employing fresh IVF showed that there was not any psychological differentiation up to the age of ten years [1]. On the other hand, progress data from undesirable pregnancies shows that seven-month-old babies presented with persistent crying and irregular biological functions. Following up on these children at the age of nine showed that they presented with aggressive behavior, while their attention was easily disrupted [43]. To conclude, it may be worth exploring the possibility that the person who takes care of the child throughout life may exert epigenetic influences on it [1].

## 5. Conclusion

Surrogacy appears to be a safe approach for certain infertility cases, presenting with promising and significant results. Most studies reveal comparable data between surrogacy and IVF cycles, as surrogacy goes hand in hand with IVF techniques. During this literature review, we attempted to isolate surrogacy data and focus on the embryo during all stages from the preimplantation to the neonatal and the period thereafter, presenting the risks entailed. It became clear that the surrogate embryo and the IVF embryo present with overlap on various concepts of management, as anticipated. This study set out to delineate and highlight the similarities and differences of a surrogate cycle embryo in comparison to the standard IVF embryo, regarding the options and at times the clinical practice. Any complications arising from the IVF practice enabling surrogacy are clearly associated with a more complex management in comparison to standard IVF cases. Surrogate embryo, fetus, neonate, and infant should be identified and examined thoroughly, as the risks related to these entities may differ. Acquiring a better understanding of what dictates these differences constitutes the base for a safer practice.
